# A case report on the management of neglected and forgotten DJ stent for 15 years with severe encrustation and multiple renal and bladder stones

**DOI:** 10.1016/j.ijscr.2022.107859

**Published:** 2022-12-31

**Authors:** Ibsa Daba Kumsa, Abeselom Lemma Gebreamlak, Messay Mekonnen Leul, Nuru Bedru Hussen, Mekbib Chere Enawgaw

**Affiliations:** Addis Ababa University, School of Medicine, Depart of Surgery, Urology Unit, PO BOX: 9086, Ethiopia

**Keywords:** CT, computerized tomography, FECal, forgotten, encrusted, calcified, PCNL, percutaneous nephrolithotomy, SWL, shock wave lithotripsy, DJ stent, Encrustation, Ureterosopy, Cytolithotrity

## Abstract

**Introduction and importance:**

The placement of ureteral DJ stents is currently regarded as a common and indispensable urologic tool (Dyer et al., 2002 [1]). However, using them can lead to complications. Infection, stent migration, encrustation, stone formation, and stent fragmentation are some of these complications (Mahmood et al., 2018 [2]). Stent-related complications are inversely associated with time (Lombardo et al., 2022 [3]).

In this case report, we present multimodal therapy, which also includes open surgery and endourologic procedures for the removal of severely encrusted DJ stents.

**Case presentation:**

A 22-year-old male who underwent nonspecific flank surgery 15 years ago, had a stent placed, and was lost to follow-up. He had severe stent encrustation at the presentation. He also had a solitary bladder stone and many pelvic stones discovered.

Initially, cytolithotrity and semirigid ureteroscopy with laser lithotripsy were performed, and the encrusted stent was removed. Subsequently, an open cytolitotomy was done. Followed by an ultrasound-guided PCNL at which time the remaining stones were removed. The patient was followed for eighteen months and has been in better condition.

**Discussion:**

The key risk factor for the development of encrustation has been shown repeatedly to be the duration of stent indwelling time (Lombardo et al., 2022 [3]). In the absence of clear guidelines for the removal of retained stents, this problem has been approached with a variety of treatment modalities (Bidnur et al., 2016 [4]). A stepwise approach with combined *endo*-urology and open surgery can be used for the management.

**Conclusion:**

Forgotten and neglected DJ stentsfor a long time can cause multiple complications. The best treatment is the prevention of this complication with a stent registry and increase awareness among the patients and their attendants.

## Highlights

• Encrustation of forgotten stents associated with large stone burden is a serious problem.
• A forgotten, encrusted & calcified stent was defined as a stent that can't be cystoscopically removed during the first try.
• To treat an encrusted stent, a progressive strategy combining endo-urology and open surgery can be used.
• The key risk factor for the development of encrustation has been shown repeatedly to be duration of stent indwelling time.

## 1 Introduction

The insertion of ureteral DJ stents is currently recognized as a common and essential urologic technique. They are used to relieve ureteral obstruction, expand ureters to ease instrumentation, prevent occlusion following procedures, and provide scaffolding for healing [3].

The most challenging complication associated with ureteral stents is encrusted and retained ureteral stents. There have been reports of complications such as irritative voiding symptoms, urinary tract obstruction, loss of renal function, serious infection, and even death. Severe encrustation can make simple office endoscopic removal difficult, necessitating surgical removal and treatment of any accompanying encrustation stones [5].

Initial attempts at removing encrusted ureteral stents are challenging. The course of action is determined by how severe the stone formations and encrustation are at the stent's two ends. In this situation, a variety of techniques have been employed to remove encrusted stents, including open surgery, percutaneous nephrolithotomy (PCNL), extracorporeal shockwave lithotripsy (SWL), cystolitholapaxy, ureteroscopic laser lithotripsy, and cystolitholapaxy [2].

In this case report, multimodal therapy is discussed for the removal of heavily encrusted DJ stents. This therapy also incorporates open surgery and endourologic procedures. To remove the encrusted stent, the patient underwent semirigid ureteroscopy and cystolithotrity. Additionally, he underwent PCNL for the lower pole renal stones and cystolithotomy for a bladder stone. These works have been reported in accordance with SCARE criteria [6].

## 2 Presentation of case

A 22-year-old man from a low socioeconomic background visited our outpatient clinic with right flank pain that had been persistent for three years and irritable urination that had gotten worse during the preceding period. A double J stent was inserted during a previous procedure he underwent in a different facility 15 years ago for stone removal. There were no medical records available. The patient was lost from follow-up because of their low socioeconomic status and low health literacy.

A physical examination revealed that he had normal vital signs and was not febrile. He had a healed surgical scar on his right flank and tenderness at the right costovertebral angle. The results of the laboratory tests were normal, and the serum creatinine level was 0.7 mg/dl. *Escherichia coli* was detected in urine culture and was treated beforehand according to sensitivity prior to the procedure.

**Fig. 1:**
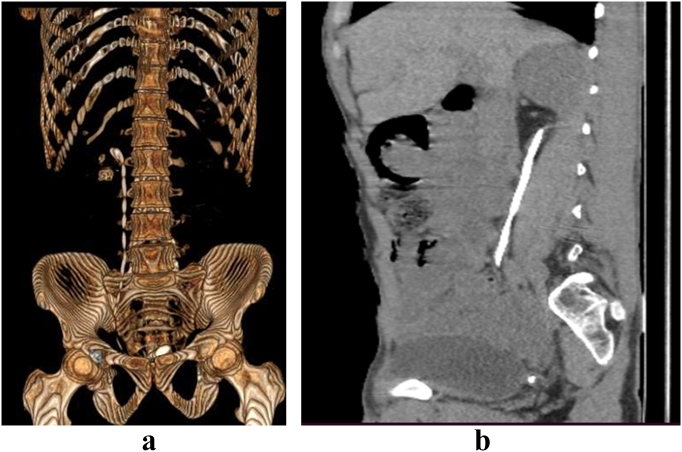
A: CT scan 3D image of Stent retained for 15 years shows significant encrustation, right nephrolithiasis and associated bladder calculus. B: CT scan sagittal view.

Preoperative abdominal and pelvic ultrasound revealed four right renal stones, the largest measuring 1.2 cm, a moderate degree of hydronephrosis, one urinary bladder stone measuring 2.7 cm, and a shadow of a double J stent. A CT scan revealed three renal pelvis stones and substantial proximal pigtail calcification with moderate degree of hydronephrosis. The bladder stonoptimizedwas 2.7 cm long, was profoundly embedded in the distal pigtail (Fig. 1).

**Fig. 2:**
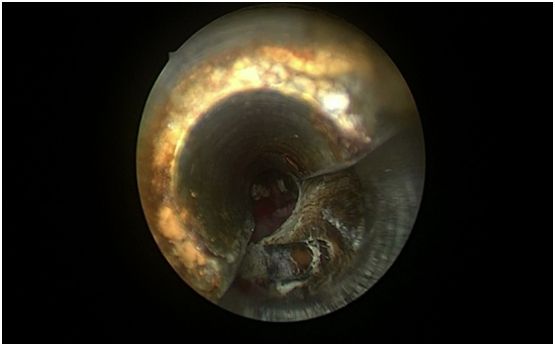
A distal coil of encrusted Double J stent being fragmented with cystolithitrity.

Multimodal therapy, endourologic treatment, and open surgery were used as therapeutic interventions. In the beginning, Maurmyers Stone Punch 25F cytolithotrity is used to remove the section of the bladder calculus along the damaged distal coil of the Double J that is broken and isolated from the proximal part (Fig. 2). The bladder residual stone remained.

**Fig. 3:**
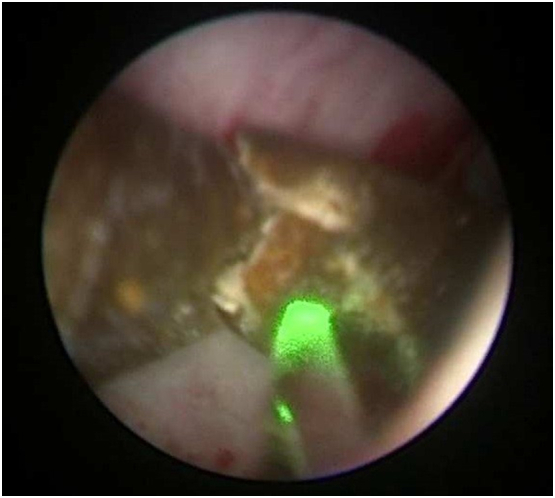
Encrusted DJ stent being fragmented with laser lithotripsy.

**Fig. 4:**
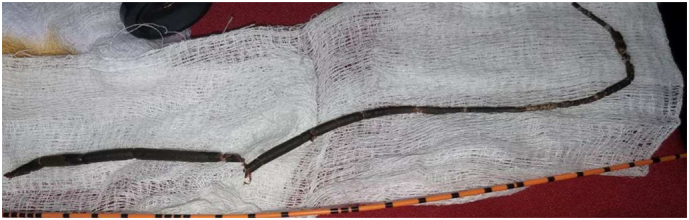
Removed encrusted DJ stent broken in two pieces. Compared to the length of ureteric catheter.

With the aid of an 8Fr Karl Storz semirigid ureteroscope and a lithotripter, a holmium (Ho: YAG) laser with a 600 um diameter has been used to disintegrate the encrustation that had grown over the proximally broken stent, and the encrusted stent was removed with grasping forceps over the course of a three-hour operation (Fig. 3, Fig. 4).

**Fig. 5:**
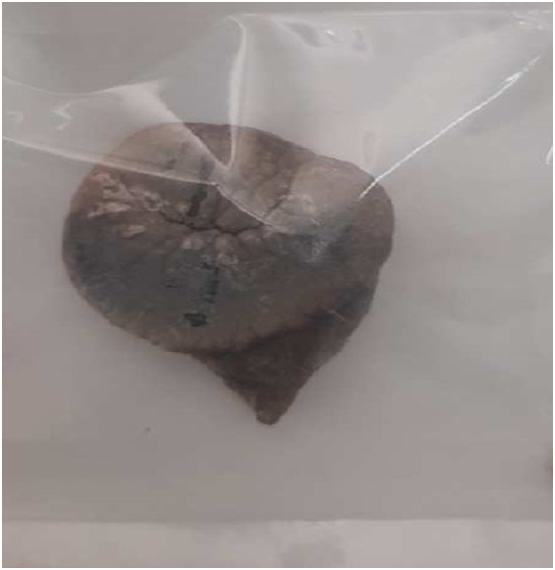
Removed badder stone with cytolithotomy.

The patient underwent an open cystolithotomy via a Pfannenstiel skin incision one week after the initial treatment, and two weeks following admission, the patient was discharged (Fig. 5). For the remaining stones in his kidney, he was readmitted once more. Following the passage of Alken metallic dilators to dilate the tract, the stones were removed using ultrasound-guided percutaneous nephrolithotomy with a standard nephroscope 24fr. Analyses of the stones revealed that they were composed of calcium oxalate.

**Fig. 6:**
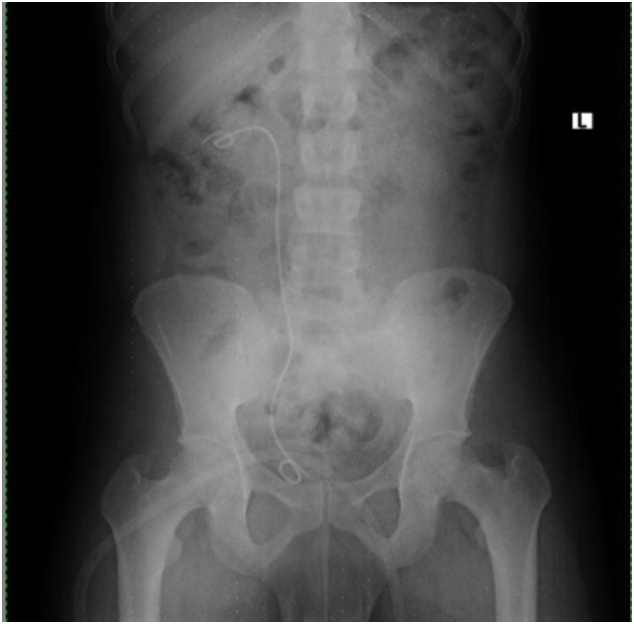
Post PCNL control KUB showing complete clearance of stone and new DJ stent.

Postoperatively, the patient underwent KUB to assess residual stone burden, and stone clearance was confirmed (Fig. 6). Outpatient follow-up in the form of a renal ultrasound showed no residual hydronephrosis. The newly inserted DJ stent was removed 3 weeks after PCNL was done.

## 3 Discussion

The insertion of ureteral stents is a crucial tool in the management of numerous urologic procedures. However, ureteral stent encrustation can result in morbidity due to infection, ureteral obstruction, and stent fragmentation [7].

An indwelling stent's surface becomes encrusted with minerals from urine deposits. Despite the fact that there are numerous risk factors for encrustation, including metabolic or congenital disorders, stone disease, bacterial colonization, chemotherapy, pregnancy, and chronic renal failure. The length of time the ureteral stent was in place before encrustation was most significant [8].

In our situation, there were numerous risk factors for stent encrustation. The stent was neglected and forgotten about for 15 years because he was not aware that it had been inserted during the initial surgery. He didn't reside close to the hospital where the initial therapy was given, which increased the rate of encrustation. He had previously undergone stone surgery. He lacks knowledge and a weak grasp of healthcare. Naturally, low patient compliance and low health literacy raise the risk of persistently retained stents [8].

The most significant pathogens for bacterial biofilm to create an encrustation include E.coli, streptococcus, and pseudomonas. In our situation, the urine culture had *E. coli*, which increases the likelihood of biofilm and encrustation forming [9].

Several grading systems exist to describe the extent of pathology and forecast surgical difficulties for stent removal after determining the extent of encrustation on imaging. According to the size, position, and degree of encrustation, the FECal system, developed by Acosta Miranda et al., and the KUB system, developed by Arenas et al., classify encrustation on a scale of 1 to 5 [10], [11].

Nir tomer, Evan Garden, and their colleagues developed a new treatment algorithm in an effort to combine the clinical usability of the FECal system treatment algorithm with the standards set forth in the KUB system. Encrustation should be examined for diameters of 5 mm or larger anytime along the stent's route using KUB, CT, or ultrasound imaging. Encrustation load is categorized as “mild” if it is less than 5 mm thick and covers less than 50 % of the stent. Cystoscopic stent removal should be tried first. When the area of encrustation exceeds 50 % of the stent and/or the encrustation burden is 5 mm or more anywhere along the stent, the pathology is deemed severe, necessitating surgical therapy. Consider extracorporeal shock wave lithotripsy as a first step if the encrustation is less than 1.5 cm, and percutaneous nephrolithotomy as the next step if it is 1.5 cm or more [3].

Similar to our patient, stents with proximal and distal encrustation, including stent fragmentation, usually require multimodal techniques to render the patient stent free. The removal of the encrusted and calcified DJ stent with PCNL, flexible URS with holmium laser, and cystolitholapaxy are the suggested treatments in this case. Due to resource constraints, combined endoscopic and open surgery was used to handle the case, which also had a successful outcome. We would like to recommend our strategy as a logical substitute for medical facilities in underdeveloped nations with little access to resources.

## 4 Conclusion

Finding ways to shorten the time that stents are in place is crucial because forgotten stents can result in serious long-term consequestudies. It is important to conduct more study on biodegradable stents, which may possibly provide financial benefits by removing the need for a second treatment and the possibility that the stents would be forgotten and retained in nations with limited resources [12].

The establishment of a national stent registry where all stent placements are registered and reminders are issued to providers to contact patients who need stent removal has been suggested by some surgeons as a way to lower the rate of forgotten stents leading to encrustation [13].

The stent should be closely watched while it is in place, removed right away when it is no longer required, and changed on a regular basis if it is required for an extended period of time. High fluid intake, prompt evaluation of clinical complaints, and aggressive treatment of infections should all be used to reduce the risk of complications [1]. Considering all of this, it is imperative to remove stents as soon as possible after they have completed their intended function in order to avoid complications and long-term morbidity.

## Consent

Written informed consent was obtained from the patient for publication of this case report and accompanying images. A copy of the written consent is available for review by the Editor-in-Chief of this journal on request.

## Ethical approval

Ethical approval was provided by the author's institution.

Ethical review committee of the Department of Surgery, College of Health Sciences, Addis Ababa University.

## Source of funding

N/A.

## Author contribution

1. Ibsa Daba,MD, Urology resident: conceived, wrote and submitted the report. Operated on the patient on both surgeries.
2. Abeselom Lemma, MD (Assistant professor of urology): Operated on the patient on the second session surgery. Reviewed the case report.
3. Messay Mekonnen, MD, General Surgeon and Assistant Professor of Urology: Operated on the patient on the first session surgery. Reviewed the case report.
4. Mekbib Chere,MD, Urologist: Operated on the patient on the first session surgery. He reviewed the case report.
5. Nuru Bedru,MD, Urology resident: Involved in incorporating the images to the paper and was involved in the management of the case.

## Guarantor

Abeselom Lemma Gebreamlak.

## Research registration number

N/A.

## Declaration of competing interest

N/A
